# Diagnostic Accuracy of Serum/Plasma Circular RNAs and the Combination of Circular RNAs and α-Fetoprotein for Detecting Hepatocellular Carcinoma: A Meta-Analysis

**DOI:** 10.3389/fgene.2021.722208

**Published:** 2021-09-30

**Authors:** Guilin Nie, Dingzhong Peng, Bei Li, Jiong Lu, Yulong Cai, Xianze Xiong, Nansheng Cheng

**Affiliations:** ^1^Department of Biliary Surgery, West China Hospital of Sichuan University, Chengdu, China; ^2^Department of Biliary Disease Research Center, West China Hospital of Sichuan University, Chengdu, China

**Keywords:** circRNA, diagnosis, biomarker, HCC, serum/plasma, meta-analysis

## Abstract

The lack of an accurate biomarker in hepatocellular carcinoma (HCC) has hindered early detection, diagnosis, and treatment. Circular RNAs (circRNAs), which can be used as novel biomarkers in liquid biopsies, have been brought to light as a result of the advances in research on molecular biomarkers and the progression of genomic medicine. We conducted a meta-analysis of the diagnostic accuracy of serum/plasma circRNAs or the combination of circRNAs and α-fetoprotein (AFP) in HCC. We identified eight studies that met the inclusion/exclusion criteria from PubMed, Web of Science, EMBASE, and Cochrane Library databases. The data were pooled, and the sensitivity, specificity, diagnostic odds ratio (DOR), positive likelihood ratio (+LR), and negative likelihood ratio (-LR) with 95% confidence intervals (CIs) were calculated. The areas under the summary receiver operator characteristic (SROC) curves (AUCs) were also calculated. The sensitivity of circRNAs was 0.82 (95% CI: 0.78–0.85), and the specificity was 0.82 (95% CI: 0.78–0.86). The sensitivity of AFP was 0.65 (95% CI: 0.61–0.68), and the specificity was 0.90 (95% CI: 0.85–0.93). The AUC was 0.89 (95% CI: 0.86–0.91) for circRNAs and 0.77 (95% CI: 0.74–0.81) for AFP. The sensitivity of the combination of circRNAs and AFP was 0.88 (95% CI: 0.84–0.92), specificity was 0.86 (95% CI: 0.80–0.91), and AUC was 0.94 (95% CI: 0.91–0.96). Additionally, a subgroup analysis was conducted based on the control groups used; the diagnostic accuracy was particularly high in the comparison of HCC vs. healthy controls. In summary, serum/plasma circRNAs are accurate biomarkers suitable for clinical use for detecting HCC, and the combination of circRNAs and AFP improved the diagnostic accuracy.

## Introduction

Hepatocellular carcinoma (HCC) is the fourth leading cause of cancer-related death worldwide. In 2020, ~905,677 new HCC cases and 830,180 deaths due to HCC were reported (Sung et al., 2021). Furthermore, about 50% of all new cases and deaths worldwide occurred in China, and HCC is one of the five leading causes of cancer-related death in China (Chen et al., 2016). Patients detected in an early stage can achieve 5-year survival to 70% with suitable therapies (Llovet et al., 1999), when the 5-year overall survival (OS) rate has reached to 21.3% until 2011 and diagnosis year (per year) (adjusted hazard ratio = 0.96) was independently associated with OS in multivariable analysis (Lee et al., 2021). Imaging methods (such as computed tomography and ultrasonography) and blood biomarkers (such as α-fetoprotein [AFP]) are usually used to screen and diagnose HCC in the clinic (Luo et al., 2018). Imaging is complex, requires sophisticated technology or partially subjective judgment (Öberg et al., 2020), and often fails to detect cases of small HCC (Luo et al., 2018). A recent meta-analysis showed that ultrasonography detected HCC with 84% sensitivity, but the sensitivity was only 47% regarding detecting early-stage HCC. However, combining ultrasonography and serum AFP improved the sensitivity of detecting of early-stage HCC to 63% (Tzartzeva et al., 2018).

Compared to radioactive or expensive imaging methods, blood biomarkers are easier to evaluate because the measurements are objective and can be obtained in real time using a relatively innocuous and low-cost venipuncture procedure (Oberg et al., 2015). However, regarding the serum biomarker AFP, its sensitivities and specificities for detecting HCC range from 39 to 64% and 76 to 91% (Oka et al., 1994; Okuda et al., 2000; Marrero and Lok, 2004), respectively. This low accuracy limits its use in clinical HCC diagnosis and asymptomatic prediction. Therefore, identifying new biomarkers for the early detection of HCC is urgent.

Endogenous circular RNAs (circRNAs) are covalently closed-loop non-coding RNAs (Jeck et al., 2013). Despite their widespread existence, only a small fraction of circRNAs have been confirmed to possess biological functions (mainly in the field of oncology) (Kristensen et al., 2019). Most functional circRNAs reportedly act as microRNA sponges (Hansen et al., 2013; Zheng et al., 2016), although others interact with proteins/mRNAs to regulate protein function (Li et al., 2015b), alter mRNA stability (Chen et al., 2019), or code proteins (Zhang et al., 2018b). CircRNAs are widely expressed in all human tissues and bodily fluids, but their functions and expression are often tissue- and developmental stage-specific (Salzman et al., 2013; Maass et al., 2017; Xia et al., 2017). These characteristics make circRNAs ideal candidates as liquid biopsy biomarkers (Wilusz, 2018; Arnaiz et al., 2019). A liquid biopsy assessment is a non-invasive method that involves using body fluids, such as blood, plasma, serum, urine, and gastric juice, to assess a disease state (Reimers and Pantel, 2019). A total of 112 differentially expressed circRNAs in body fluids had been identified in various cancers up to May 15, 2020 (Wang et al., 2021).

In our meta-analysis, we analyzed the diagnostic accuracy of serum/plasma circRNAs for detecting HCC based on the results of eight studies published up to May 27, 2021. The diagnostic accuracies of circRNAs, AFP, and the combination of both were compared based on the pooled sensitivity, specificity, area under the summary receiver operator characteristic (SROC) curve (AUC), and other statistical indicators. Additionally, the pooled values were compared between HCC patients and various control groups (healthy controls, cirrhosis patients, hepatitis patients, and non-HCC patients) to assess whether the diagnostic accuracy differed.

## Methods

### Search Strategy

Two investigators (Peng DZ and Li B) searched for relevant studies in PubMed, Web of Science, Cochrane Library, and EMBASE up to May 27, 2021. The search strategy involved the following keywords: (“circRNA or circular RNA”) and (“AFP or α-fetoprotein”) and (“plasma or serum”) and (“liver cancer or liver carcinoma or hepatocellular carcinoma or HCC”). We also searched the references of these articles and contacted the authors for more details when necessary.

### Study Selection Criteria

The study inclusion criteria were as follows: (1) explored the relationships of circRNAs and AFP in the serum/plasma with HCC; (2) HCC was diagnosed based on histopathology; (3) case–control or cohort study; and (4) contained adequate information so that true positives (TP), true negatives (TN), false positives (FP), and false negatives (FN) could be calculated regarding HCC diagnosis. The study exclusion criteria were as follows: (1) letters, case reports, meta-analyses, reviews, or animal studies; (2) not relevant to circRNAs and AFP in the serum/plasma and HCC; (3) unavailable or incomplete data; and (4) written in other languages instead of English.

### Data Extraction and Quality Assessment

Two investigators (Peng DZ and Li B) independently extracted the following data: (1) first author, publication year, type of cancer and circRNA, specimen type and size, circRNA assay method, and study location; (2) TP, TN, FP, FN, and AUC for diagnostic analysis; and (3) circRNA expression levels, AFP levels. The quality of the included studies was evaluated using Quality Assessment of Diagnostic Accuracy Studies 2 (QUADAS-2) (Whiting et al., 2011). A third investigator (Cai YL) resolved any disagreements.

### Statistical Analysis

The data were analyzed using STATA v15.0 and Review Manager v5.4. The pooled sensitivity and specificity (with 95% CIs) were calculated to determine the diagnostic accuracy of the biomarkers. Pooled diagnostic odds ratio (DOR), positive likelihood ratio (+LR), and negative likelihood ratio (–LR) were calculated. Summary receiver operator characteristic (SROC) curves were plotted, and the AUCs were calculated. The threshold effect was assessed by correlation coefficient and p-value. *p* < 0.05 indicated the existence of threshold effect. Considering the between-study variation in thresholds, we used the hierarchical summary receiver operating characteristics (HSROC) to compare the SROC curve (Rutter and Gatsonis, 2001). Heterogeneity was estimated using the I^2^ value and Cochran's *Q* test (Higgins et al., 2003), with I^2^ > 50% and *p* < 0.05 suggesting significant heterogeneity. A random-effect model was applied due to significant heterogeneity. A subgroup-analysis was processed with a high heterogeneity. Publication bias was evaluated using Deek's funnel plot asymmetry test. *p* < 0.10 for the slope coefficient indicated significant asymmetry and the existence of publication bias.

## Results

### Included Studies

**Figure 1:**
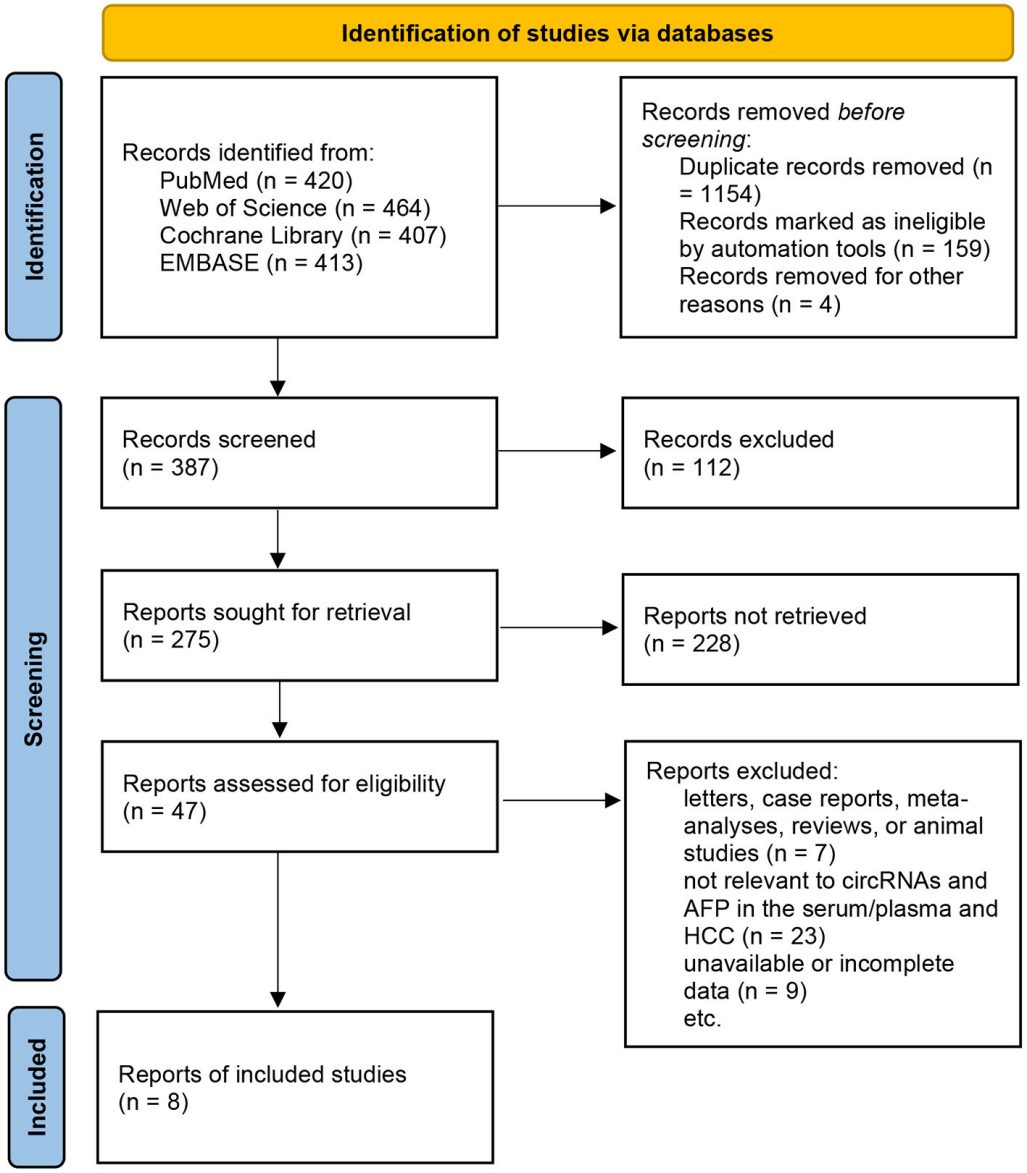
Flow-process diagram of the study selection process.

Eight studies were included in this review (Zhang et al., 2018c,d; Li et al., 2019; Qiao et al., 2019; Wu et al., 2020; Yu et al., 2020; Zhu et al., 2020; Liu et al., 2021), and the sample sizes ranged from 156 to 540 patients. All blood samples included in the studies were examined for the levels of circRNAs and AFP. All the studies included sufficient data for the calculation of sensitivity, specificity, and other statistical indicators. The details are shown in Supplementary Tables 1–3; Figure 1.

### Primary Meta-Analysis

**Figure 2:**
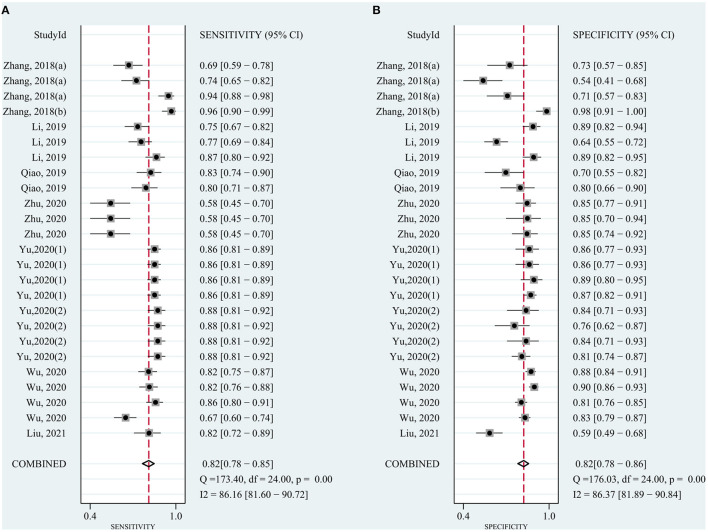
Forest plots for diagnostic accuracy of circRNAs in hepatocellular carcinoma (HCC). **(A)** Sensitivity. **(B)** Specificity.

**Figure 3:**
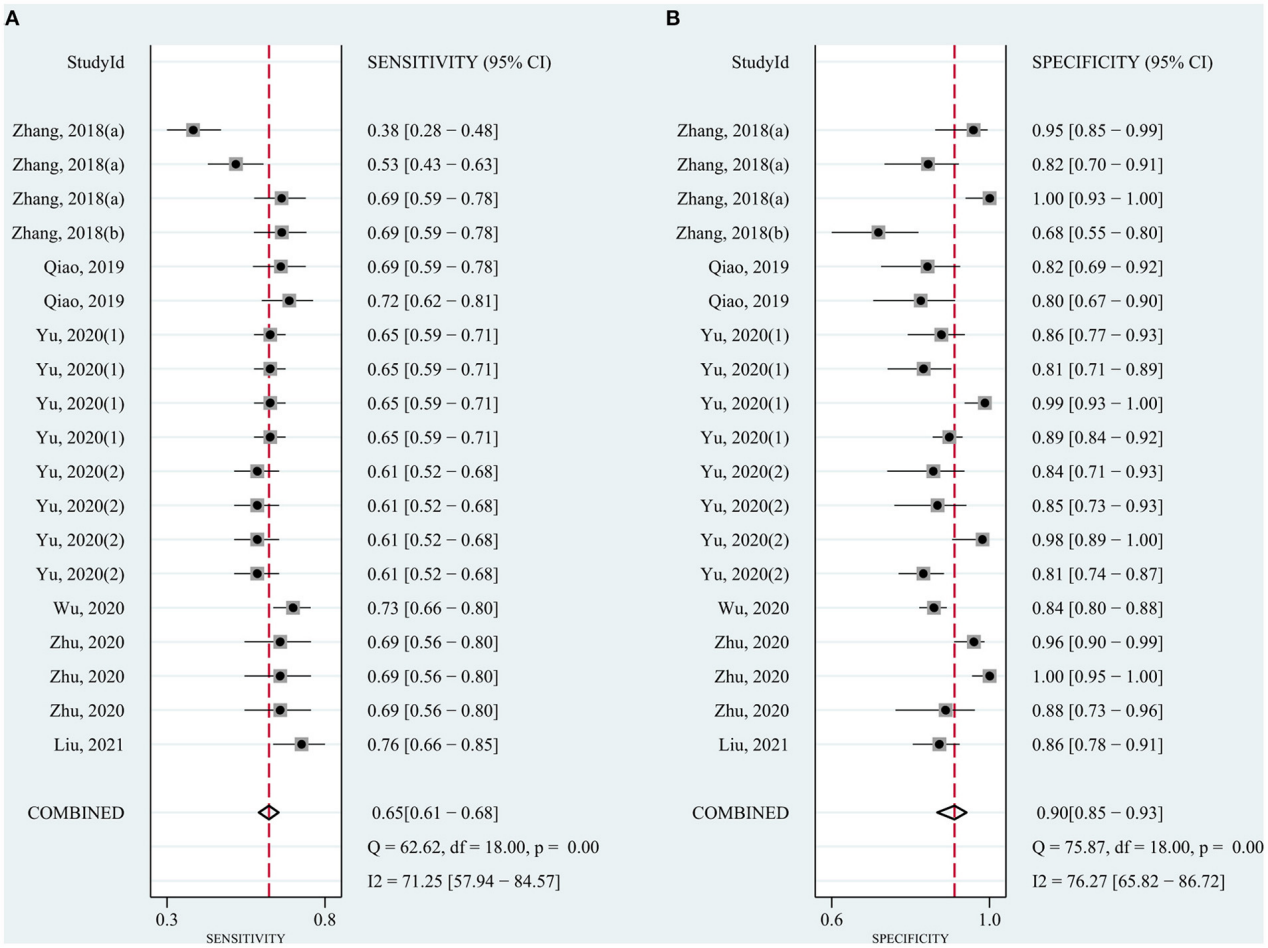
Forest plots for diagnostic accuracy of AFP in hepatocellular carcinoma (HCC). **(A)** Sensitivity. **(B)** Specificity.

The pooled sensitivity was 0.82 (95% CI: 0.78–0.85) for circRNAs and 0.65 (95% CI: 0.61–0.68) for AFP, and the pooled specificity was 0.82 (95% CI: 0.78–0.86) and 0.90 (95% CI: 0.85–0.93), respectively. Cochran's Q was 173.40–176.03 (*p* < 0.001) for circRNAs and 62.62–75.87 (*p* < 0.001) for AFP, and I^2^ was 86% and 71–76%, respectively, indicating moderate heterogeneity (Figures 2, 3). The correlation coefficient of the threshold effect of circRNAs was 0.26 (*p* = 0.07), which meant no influence of threshold effect. The correlation coefficient of AFP was −0.20 (*P* = 0.04).

**Figure 4:**
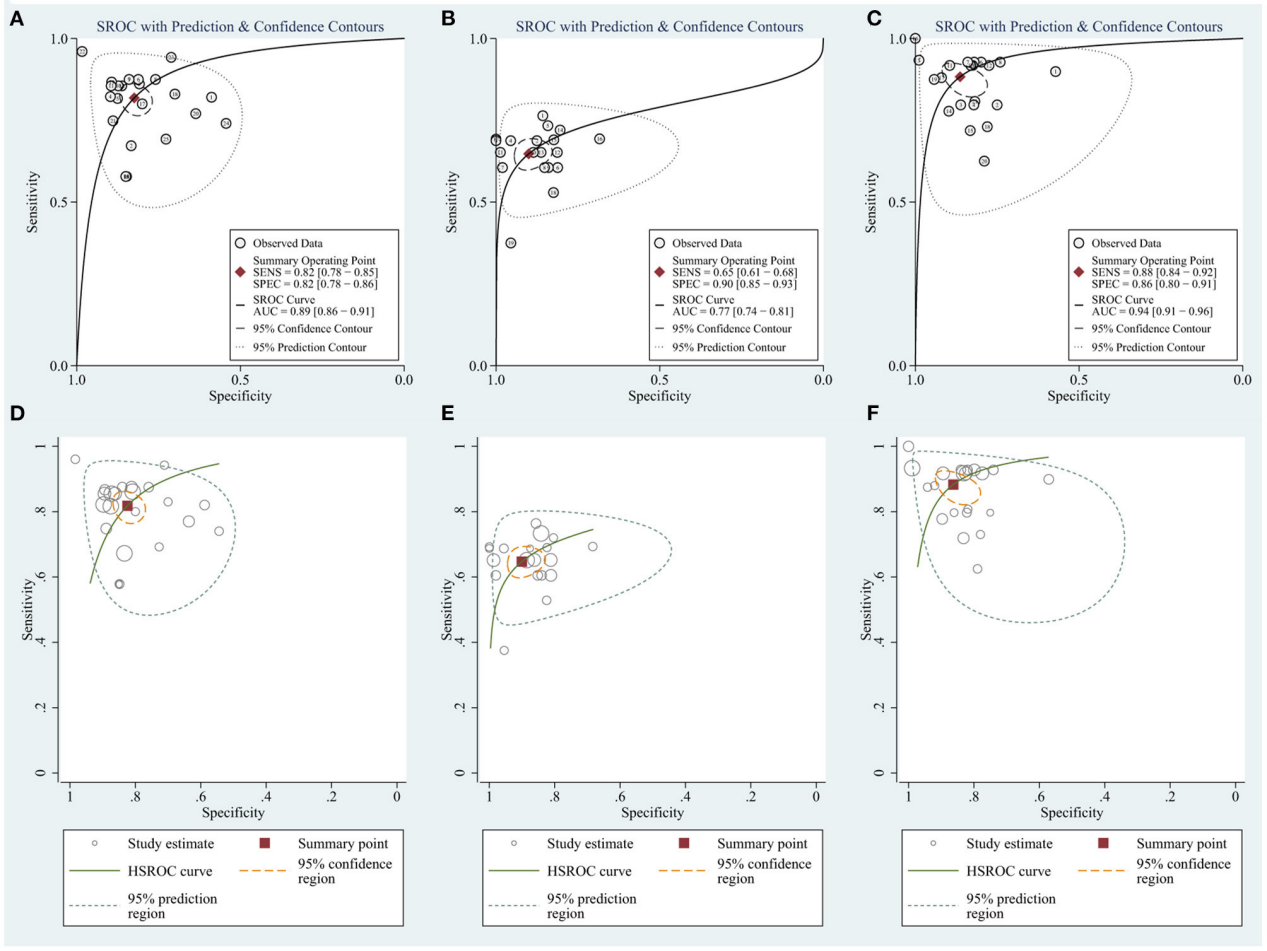
The summary receiver operator characteristic (SROC) for **(A)** circRNAs. **(B)** AFP. **(C)** Combination of circRNAs and AFP and the hierarchical summary receiver operating characteristics (HSROC) for **(D)** circRNAs. **(E)** AFP. **(F)** Combination of circRNAs and AFP.

To evaluate the value of the biomarkers for detecting HCC, a meta-analysis of the AUCs from the eight studies was conducted. The pooled AUC was 0.89 (95% CI: 0.86–0.91) for circRNAs (Figure 4A) and 0.77 (95% CI: 0.74–0.81) for AFP (Figure 4B). Cochran's Q was 61.54 (*p* < 0.001) for circRNAs and 40.37 (*p* < 0.001) for AFP, respectively, and I^2^ was 97% for circRNAs and 95% for AFP, indicating considerable heterogeneity. Meanwhile, HSROC compared SROC curves with between-study variation in thresholds (Figures 4D,E).

In summary, circRNAs demonstrate higher diagnostic accuracy compared to AFP, especially regarding sensitivity.

### Subgroup Analysis

The significant heterogeneity prompted us to a subgroup analysis. It was divided into four groups—HCC vs. healthy controls, HCC vs. cirrhosis patients, HCC vs. hepatitis patients, and HCC vs. non-HCC patients (based on the control groups in the included studies). The sensitivity, specificity, AUCs, and other statistics were calculated and compared (Table 1).

**Table 1:** Subgroup analysis of diagnostic accuracy.

	**No**.	**Sen**	**Spe**	**DOR**	**+LR**	**-LR**	**AUC**	**Heterogeneity**	
		**(95% CI)**	**(95% CI)**	**(95% CI)**	**(95% CI)**	**(95% CI)**	**(95% CI)**	**I^2^**	**p**
**HCC vs. healthy controls**									
circRNAs	7	0.87 (0.78–0.92)	0.87 (0.80–0.92)	43 (17–109)	6.6 (4.1–10.8)	0.15 (0.09–0.27)	0.93 (0.91–0.95)	84	0.001
AFP	6	0.67 (0.63–0.70)	0.98 (0.85–1.00)	78 (12–534)	26.8 (4.0–179.9)	0.34 (0.31–0.38)	0.70 (0.66–0.74)	96	0.000
Combination	6	0.93 (0.82–0.98)	0.93 (0.84–0.97)	194 (26–1456)	13.7 (5.1–36.7)	0.07 (0.02–0.22)	0.98 (0.96–0.99)	0	0.425
**HCC vs. cirrhosis**									
circRNAs	5	0.78 (0.69–0.86)	0.74 (0.62–0.84)	10 (5–23)	3.0 (1.9–4.8)	0.29 (0.19–0.45)	0.83 (0.80–0.86)	73	0.012
AFP	4	0.62 (0.57–0.67)	0.85 (0.80–0.89)	9 (6–15)	4.1 (2.9–5.8)	0.45 (0.38–0.52)	0.84 (0.80–0.87)	0	0.491
Combination	5	0.83 (0.70–0.91)	0.81 (0.77–0.85)	21 (9–26)	4.4 (3.4–5.8)	0.21 (0.12–0.39)	0.82 (0.79–0.85)	88	0.000
**HCC vs. hepatitis**									
circRNAs	5	0.81 (0.74–0.86)	0.82 (0.74–0.88)	19 (10–36)	4.5 (3.1–6.6)	0.23 (0.17–0.32)	0.88 (0.85–0.91)	33	0.112
AFP	4	0.58 (0.46–0.69)	0.87 (0.77–0.93)	9 (6–15)	4.5 (2.8–7.1)	0.48 (0.38–0.60)	0.81 (0.77–0.84)	87	0.000
Combination	5	0.85 (0.77–0.91)	0.82 (0.76–0.87)	26 (17–40)	4.7 (3.6–6.1)	0.18 (0.12–0.28)	0.89 (0.86–0.91)	90	0.000
**HCC vs. non-HCC**									
circRNAs	8	0.80 (0.74–0.85)	0.83 (0.77–0.88)	20 (12–33)	4.7 (3.5–6.4)	0.24 (0.18–0.32)	0.89 (0.86–0.91)	94	0.000
AFP	5	0.69 (0.63–0.73)	0.87 (0.82–0.91)	15 (9–24)	5.4 (3.8–7.7)	0.36 (0.30–0.43)	0.82 (0.79–0.85)	0	0.121
Combination	5	0.91 (0.87–0.93)	0.86 (0.65–0.95)	61 (15–244)	66 (2.3–18.7)	0.11 (0.07–0.16)	0.93 (0.90–0.95)	93	0.000
**Overall**									
circRNAs	25	0.82 (0.78–0.85)	0.82 (0.78–0.86)	21 (14–31)	4.7 (3.7–5.8)	0.22 (0.18–0.27)	0.89 (0.86–0.91)	97	0.000
AFP	19	0.65 (0.61–0.68)	0.90 (0.85–0.93)	17 (10–26)	6.5 (4.3–9.7)	0.39 (0.35–0.43)	0.77 (0.74–0.81)	95	0.000
Combination	21	0.88 (0.84–0.92)	0.86 (0.80–0.91)	48 (24–94)	6.5 (4.4–9.5)	0.14 (0.10–0.19)	0.94 (0.91–0.96)	97	0.000

*Sen, sensitivity; Spe, specificity; +LR, positive likelihood ratio; –LR, negative likelihood ratio; DOR, diagnostic OR; AUC, area under the curve; HCC, hepatocellular carcinoma; AFP, α-fetoprotein*.

#### Subgroup Analysis of HCC Patients vs. Healthy Controls

##### circRNAs

Six studies compared circRNAs between HCC patients and healthy controls. The sensitivity was 0.87 (95% CI: 0.78–0.92), and specificity was 0.87 (95% CI: 0.80–0.92) (Supplementary Figure 2A). The AUC was 0.93 (95% CI: 0.91–0.95), and I^2^ was 84% (*p* = 0.001). The DOR was 43 (95% CI: 17–109), +LR was 6.6 (95% CI: 4.1–10.8), and –LR was 0.15 (95% CI: 0.09–0.27).

##### AFP

Five studies compared AFP between HCC patients and healthy controls. The sensitivity was 0.67 (95% CI: 0.63–0.70), and specificity was 0.98 (95% CI: 0.85–1.00) (Supplementary Figure 2E). The AUC was 0.70 (95% CI: 0.66–0.74), and I^2^ was 96% (*p* < 0.001). The DOR was 78 (95% CI: 12–534), +LR was 26.8 (95% CI: 4.0–179.9), and –LR was 0.34 (95% CI: 0.31–0.38).

#### Subgroup Analysis of HCC vs. Cirrhosis Patients

##### circRNAs

Four studies compared circRNAs between HCC and cirrhosis patients. The sensitivity was 0.78 (95% CI: 0.69–0.86), and specificity was 0.74 (95% CI: 0.62–0.84) (Supplementary Figure 2B). The AUC was 0.83 (95% CI: 0.80–0.86), and I^2^ was 73% (*P* = 0.012). The DOR was 10 (95% CI: 5–23), +LR was 3.0 (95% CI: 1.9–4.8), and –LR was 0.29 (95% CI: 0.19–0.45).

##### AFP

Three studies compared AFP between HCC and cirrhosis patients. The sensitivity was 0.62 (95% CI: 0.57–0.67), and specificity was 0.85 (95% CI: 0.80–0.89) (Supplementary Figure 2F). The AUC was 0.84 (95% CI: 0.80–0.87), and there was no heterogeneity (*I*^2^ = 0; *p* = 0.491). The DOR was 9 (95% CI: 6–15), +LR was 4.1 (95% CI: 2.9–5.8), and -LR was 0045 (95% CI: 0.38–0.52).

#### Subgroup Analysis of HCC vs. Hepatitis Patients

##### circRNAs

Four studies compared circRNAs between HCC and hepatitis patients. The sensitivity was 0.81 (95% CI: 0.74–0.86), and specificity was 0.82 (95% CI: 0.74–0.88) (Supplementary Figure 2C). The AUC was 0.88 (95% CI: 0.85–0.91), and I^2^ was 33% (*p* = 0.112). The DOR was 19 (95% CI: 10–36), +LR was 4.5 (95% CI; 3.1–6.6), and –LR was 0.23 (95% CI: 0.17–0.32).

##### AFP

Three studies compared AFP between HCC and hepatitis patients. The sensitivity was 0.58 (95% CI: 0.46–0.69), and specificity was 0.87 (95% CI: 0.77–0.93) (Supplementary Figure 2G). The AUC was 0.81 (95% CI: 0.77–0.84), and I^2^ was 87% (*p* < 0.001). The DOR was 9 (95% CI: 6–15), +LR was 4.5 (95% CI: 2.8–7.1), and –LR was 0.48 (95% CI: 0.38–0.60).

#### Subgroup Analysis of HCC vs. Non-HCC Patients

##### circRNAs

Four studies compared circRNAs between HCC and non-HCC patients. The sensitivity was 0.80 (95% CI: 0.74–0.85) and specificity was 0.83 (95% CI: 0.77–0.88) (Supplementary Figure 2D). The AUC was 0.89 (95% CI: 0.86–0.91), and I^2^ was 94% (*p* < 0.001). The DOR was 20 (95% CI: 12–33), +LR was 4.7 (95% CI: 3.5–6.4), and –LR was 0.24 (95% CI: 0.18–0.32).

##### AFP

Four studies compared AFP between HCC and non-HCC patients. The sensitivity was 0.69 (95% CI: 0.63–0.73), and specificity was 0.87 (95% CI: 0.82–0.91) (Supplementary Figure 2H). The AUC was 0.82 (0.79–0.85), and I^2^ was 30% (*p* = 0.121). The DOR was 15 (95% CI: 9–24), +LR was 5.4 (95% CI: 3.8–7.7), and –LR was 0.36 (95% CI: 0.30–0.43).

### Combination of circRNAs and AFP

**Figure 5:**
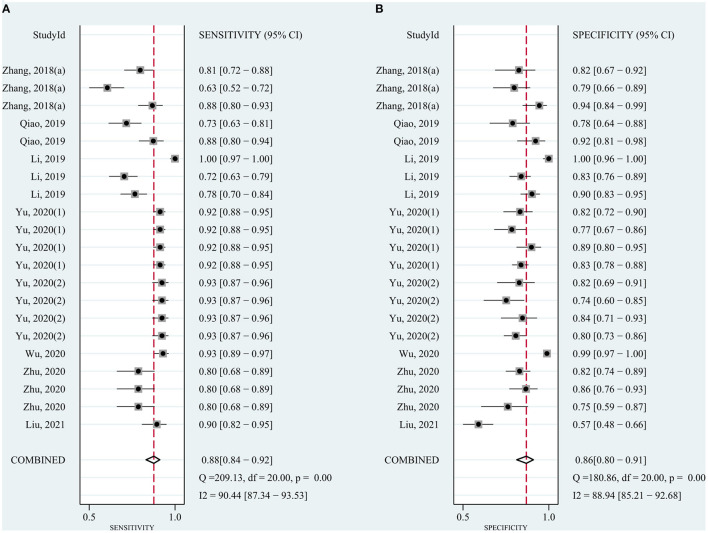
Forest plots for sensitivity and specificity of the combination of circRNAs and AFP in HCC.

Considering the widespread use and fair performance of AFP, we assessed the diagnostic accuracy of the combination of circRNAs and AFP for detecting HCC. Six studies assessed the diagnostic accuracy of the combination of circRNAs and AFP. The sensitivity was 0.88 (95% CI: 0.84–0.92), specificity was 0.86 (95% CI: 0.80–0.91) (Figure 5), and AUC was 0.94 (95% CI: 0.91–0.96) (Figure 4C). Cochran's Q was 58.88 (*p* < 0.001), and I^2^ was 97%. The correlation coefficient of threshold effect of the combination was 0.53 (*p* = 0.28). The HSROC was showed in Figure 4F.

Regarding the subgroups, the sensitivities regarding the comparisons with healthy controls, cirrhosis patients, hepatitis patients, and non-HCC patients were 0.93 (95% CI: 0.82–0.98), 0.83 (95% CI: 0.70–0.91), 0.85 (95% CI: 0.77–0.91), and 0.91 (95% CI: 0.87–0.93), respectively. The specificities were 0.93 (95% CI: 0.84–0.97), 0.81 (95% CI: 0.77–0.85), 0.82 (95% CI: 0.76–0.87), and 0.86 (95% CI: 0.65–0.95), respectively. The AUCs were 0.98 (95% CI: 0.96–0.99; *I*^2^ = 0, *P* = 0.425), 0.82 (95% CI: 0.79–0.85; *I*^2^ = 88%, *P* < 0.001), 0.89 (95% CI: 0.86–0.91; *I*^2^ = 90%, *P* < 0.001), and 0.93 (95% CI: 0.90–0.95; *I*^2^ = 93%, *P* < 0.001), respectively.

### Publication Bias

**Figure 6:**
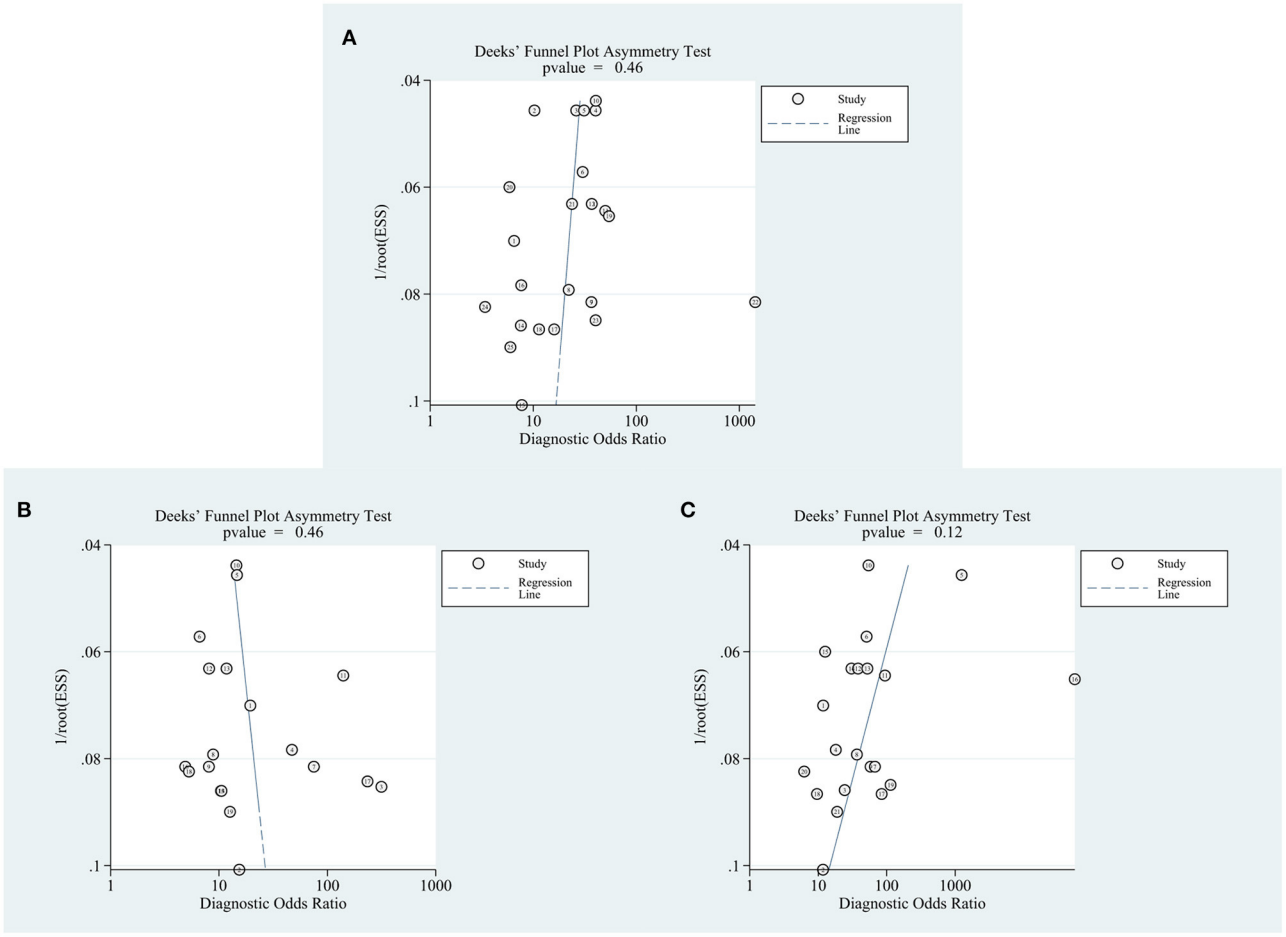
Forest plots for publication bias. **(A)** circRNAs. **(B)** AFP. **(C)** Combination of circRNAs and AFP.

Regarding the analyses of the diagnostic accuracy of circRNAs, AFP, and the combination of both, the *p* values of Deek's funnel plot asymmetry test were 0.46, 0.46, and 0.12, respectively, indicating a low likelihood of publication bias (Figure 6).

## Discussion

The high mortality rate of HCC, which ideally should be considerably lower, is partly due to the absence of early warning symptoms and the poor performance of blood biomarkers, such as AFP and other potential blood biomarkers, e.g., glypican-3 (GPC3), des-γ-carboxyprothrombin (DCP), and microRNAs (Yu et al., 2020). It has been reported that the sensitivities and specificities of AFP in the blood for identifying HCC ranged from 39 to 64% and 76 to 91% (Oka et al., 1994; Marrero and Lok, 2004; Luo et al., 2018), respectively. In this meta-analysis, the sensitivity and specificity were 65% and 90%, and the AUC was 0.77. It has been reported that the sensitivities and specificities of GPC3 were 71–84% and 65–100% (Waidely et al., 2016). According to a meta-analysis in 2014, the sensitivity and specificity of DCP were 71 and 84% for detecting HCC (Zhu et al., 2014). In another meta-analysis in 2018, the sensitivity and specificity were 71 and 93% for detecting HBV-related HCC (Chen et al., 2018). A multicenter retrospective study showed that the sensitivities and specificities of Cmi (a combination of seven blood microRNAs) for detecting HCC in various cohorts were 70–86% and 80–91%, respectively (Lin et al., 2015). Additionally, the GALAD score, which comprises two demographic risk factors (gender and age) and three tumor markers (AFP, AFP-L3, and DCP), performed well in detecting early-stage HCC (Johnson et al., 2014). Moreover, a meta-analysis reported that the sensitivity and specificity of miRNAs in the blood (single miRNAs or miRNA panels) regarding discriminating HCC from non-HCC patients were 81 and 76% (Peng et al., 2019). Despite this, more accurate (highly sensitive and specific) novel blood biomarkers are urgently needed for the detection of HCC, especially early-stage or small HCC.

In previous studies, blood circRNAs exhibited valuable performance as diagnostic biomarkers for various cancers, including HCC (Qiu et al., 2019), prostate cancer (Vo et al., 2019), lung cancer (Hang et al., 2018), gastric cancer (Tang et al., 2018), and pancreatic cancer (Rong et al., 2021), partly due to its characteristic structure and unclear exocytosis (Li et al., 2018). In the studies included in our meta-analysis, the AUCs for circRNAs ranged from 0.64 to 0.95, with sensitivities of 58 to 94% and specificities of 54 to 98%. Based on the pooled AUC (0.89), sensitivity (0.82), and specificity (0.82) for circRNAs, and the corresponding results for AFP (0.77, 0.65, and 0.90), serum/plasma circRNAs are more suitable biomarkers for the early detection of HCC than AFP, especially regarding sensitivity. However, regarding the pooled specificity, AFP performed better than circRNAs.

The subgroup analysis compared HCC vs. healthy controls, HCC vs. cirrhosis patients, HCC vs. hepatitis patients, and HCC vs. non-HCC patients (based on the control groups in the included studies). In all four subgroups, circRNAs were more sensitive than AFP (0.78–0.87 vs. 0.67–0.69) and had superior AUCs (0.83–0.93 vs. 0.70–0.82).

In detail, the subgroup analysis showed that circRNAs were particularly effective in distinguishing HCC patients and healthy controls (sensitivity: 0.87; specificity: 0.87; AUCs: 0.93). Meanwhile, in the same subgroup, AFP showed a better specificity (0.98) but a lower sensitivity (0.67) and AUCs (0.70). These results indicated that circRNAs had a more effective diagnostic efficiency in reckoning both specificity and sensitivity. Besides, with a high sensitivity, circRNAs were more suitable selections for early detection and surveillance biomarkers in healthy people.

However, circRNAs were less good at distinguishing HCC and cirrhosis patients (sensitivity: 0.78; specificity: 0.74; AUC: 0.83). In the HCC vs. cirrhosis analysis, circRNAs were similar to AFP regarding AUC (0.83 vs. 0.84), superior regarding sensitivity (0.78 vs. 0.62), and inferior regarding specificity (0.74 vs. 0.85). Similar results appeared in group HCC vs. hepatitis. Notably, the sensitivity of AFP is particularly low in both groups (0.62 and 0.58). These results indicate that although individual or multiple circRNAs are not perfect diagnostic biomarkers, and showed some limitations, they could be used as screening tools on the basis of extra higher sensitivity than AFP. Correspondingly, the possibility about the combination of circRNAs and imaging methods to early detect HCC needs to be explored. One of the included multicenter studies reported that the diagnostic accuracy (including sensitivity and specificity) of CircPanel was superior for diagnosing HCC, small-HCC (solitary, diameter ≤ 3 cm), or AFP-negative HCC with cirrhosis or hepatitis (Yu et al., 2020). Therefore, more studies are urged to solve the complexity and uncertainty of circRNAs.

When it came to HCC and non-HCC patients, the low sensitivity (0.69), low AUCs (0.82), and relatively insufficient specificity (0.87) of AFP showed its restricted roles in screening and diagnosing HCC from non-HCC people. By contraries, considering the high sensitivity and AUCs, circRNAs might be worthier serum/plasma biomarkers for non-HCC people. Additionally, two studies explored the difference between preoperative and postoperative circRNAs, and results were as expected (Zhang et al., 2018c; Wu et al., 2020). These studies highlight that the detailed roles of serum/plasma circRNAs, such as in early HCC, micro-metastasis, or HCC recurrence, should be identified to ensure their maximum usefulness.

In 2015, Li et al., for the first time, reported the presence of abundant exosomal circRNAs and explored their potential diagnostic accuracy (Li et al., 2015a). Thereafter, Zhang et al. reported the difference in lnRNA-HEIH levels between serum and serum exosomes (Zhang et al., 2018a). However, the differences in circRNAs between serum/plasma and serum/plasma exosomes, or between extracellular vesicles of varying diameters, remain unclear. Besides serum/plasma circRNAs, circ_0000798 extracted from peripheral blood mononuclear cells had an AUC of 0.70 (95% CI: 0.60–0.80) for distinguishing HCC patients from healthy controls (Lei et al., 2019). Wang et al. reported that circSATD3 in peripheral venous blood could predict microvascular invasion with an AUC of 0.637 (95% CI: 0.529–0.755) (Wang et al., 2020). Therefore, more studies are needed to determine the diagnostic accuracy of circRNAs in the different components of blood for detecting HCC.

We also examined the diagnostic accuracy of the combination of circRNAs and AFP and found that it was remarkably improved. The AUC was 0.94 (95% CI: 0.91–0.96), with a high sensitivity (0.88) and specificity (0.86); the AUCs were superior to the AUCs of a combination of tissue and blood parameters (0.84) in a meta-analysis in 2019 (Huang et al., 2019). In our subgroup analysis, the diagnostic accuracy of the combination of circRNAs and AFP was extremely high in HCC vs. healthy controls, with a sensitivity, specificity, and AUC of 0.93, 0.93, and 0.98 (*I*^2^ = 0). In contrast, the combination of circRNAs and AFP was relatively inaccurate in discriminating HCC patients from cirrhosis patients. In general, the excellent performance of the combination of circRNAs and AFP should prompt large-scale multicenter studies to be conducted to develop various models to distinguish HCC from various conditions or to develop an optimal universal model. Of course, it remains unclear which are more effective between single- and multi-circRNA models.

Limitations inevitably exist in our meta-analysis. First, the data all come from China, which somewhat limits the generalizability of our conclusions. This phenomenon may be caused by the fact that sufficient research funding was invested in this area as a result of the high incidence of HCC in China. Second, the level of the evidence was low, as most research has only reached phase 3 (retrospective longitudinal studies) rather than phase 4 (prospective screening studies) or 5 (cancer control studies, which determine whether surveillance of an at-risk population using the novel biomarker reduces the cancer burden compared to no surveillance) (Marrero and Lok, 2004). Third, the comparatively poor performance of circRNAs or the combination of circRNAs and AFP in distinguishing HCC patients from patients with cirrhosis or hepatitis necessitates identifying more suitable circRNAs or models for screening, as the goal of HCC surveillance program is to diagnose and treat HCC early in *high-risk* individuals to improve long-term outcomes (Yang and Kim, 2012). High-risk individuals are defined as Asian men (age >40 years) or Asian women (age >50 years), individuals with a family history of HCC, and African or African-American individuals, all with hepatitis B or individuals with cirrhosis, by the American Association for the Study of Liver Diseases (AASLD) and Asian Pacific Association for the Study of the Liver (APASL) (Omata et al., 2017; Marrero et al., 2018). Lastly, high heterogeneity still existed in some subgroups.

In conclusion, the meta-analysis revealed the remarkable diagnostic accuracy of circRNAs extracted from serum/plasma for detecting HCC. The combination of circRNAs and AFP improved the diagnostic accuracy. This discovery reminds us of the benefit of combining circRNAs and other biomarkers or using multiple circRNAs. Additionally, diagnostic accuracy varied among subgroups and was highest in the comparison of HCC patients vs. healthy controls.

## Data Availability Statement

The original contributions presented in the study are included in the article/Supplementary Material, further inquiries can be directed to the corresponding author/s.

## Author Contributions

NC and XX: conception and design. NC: administrative support. BL, YC, and DP: collection and assembly of data. GN and JL: data analysis and interpretation. GN: manuscript writing. All authors contributed to the article and approved the submitted version.

## Funding

This study was supported by the National Natural Science Foundation of China (Grant No. 81900516) and Science and Technology Support Project of Sichuan Province (Grant Nos. 2020YSF0238 and 2019YFS0041).

## Conflict of Interest

The authors declare that the research was conducted in the absence of any commercial or financial relationships that could be construed as a potential conflict of interest.

## Publisher's Note

All claims expressed in this article are solely those of the authors and do not necessarily represent those of their affiliated organizations, or those of the publisher, the editors and the reviewers. Any product that may be evaluated in this article, or claim that may be made by its manufacturer, is not guaranteed or endorsed by the publisher.
