# Supplementary material for: Diagnostic Accuracy of Serum/Plasma Circular RNAs and the Combination of Circular RNAs and α-Fetoprotein for Detecting Hepatocellular Carcinoma: A Meta-Analysis
Source: Front Genet. 2021 Sep 30;12:722208. doi: 10.3389/fgene.2021.722208 (PMC8514948; doi:10.3389/fgene.2021.722208)
Supplement: Supplementary file 1 [file Data_Sheet_1.zip › Sup Figure.DOCX]

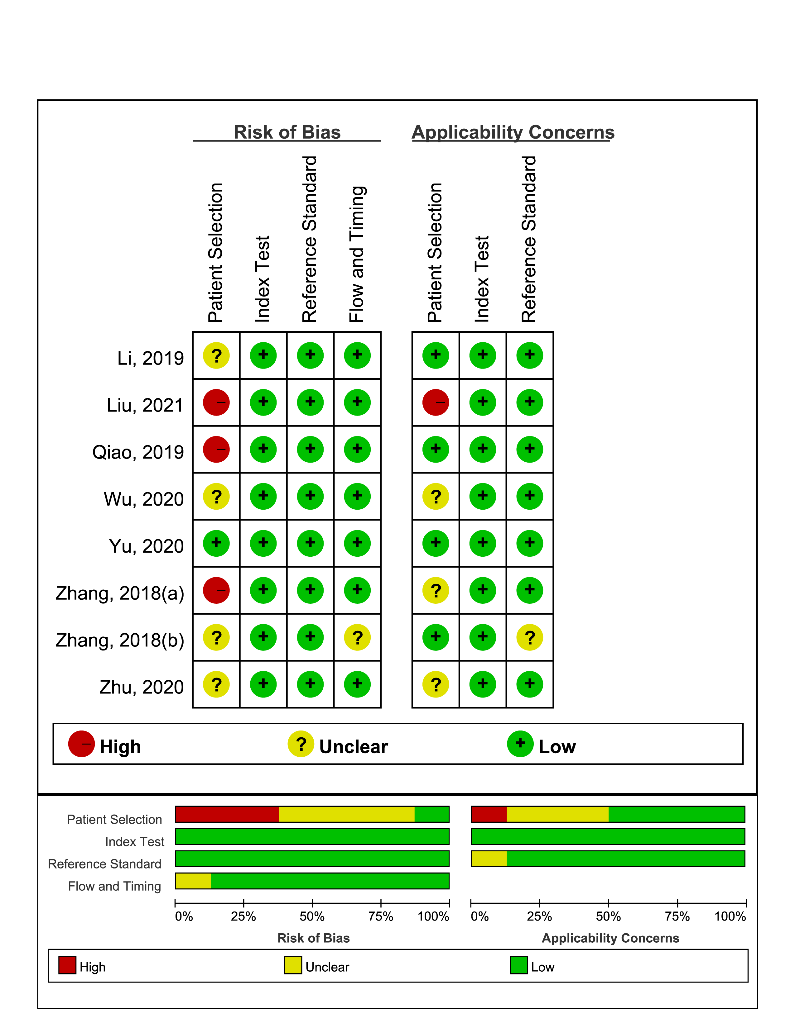
**Supplementary Figure 1:** **Methodological quality of the included studies (QUADAS-2 results).**

**
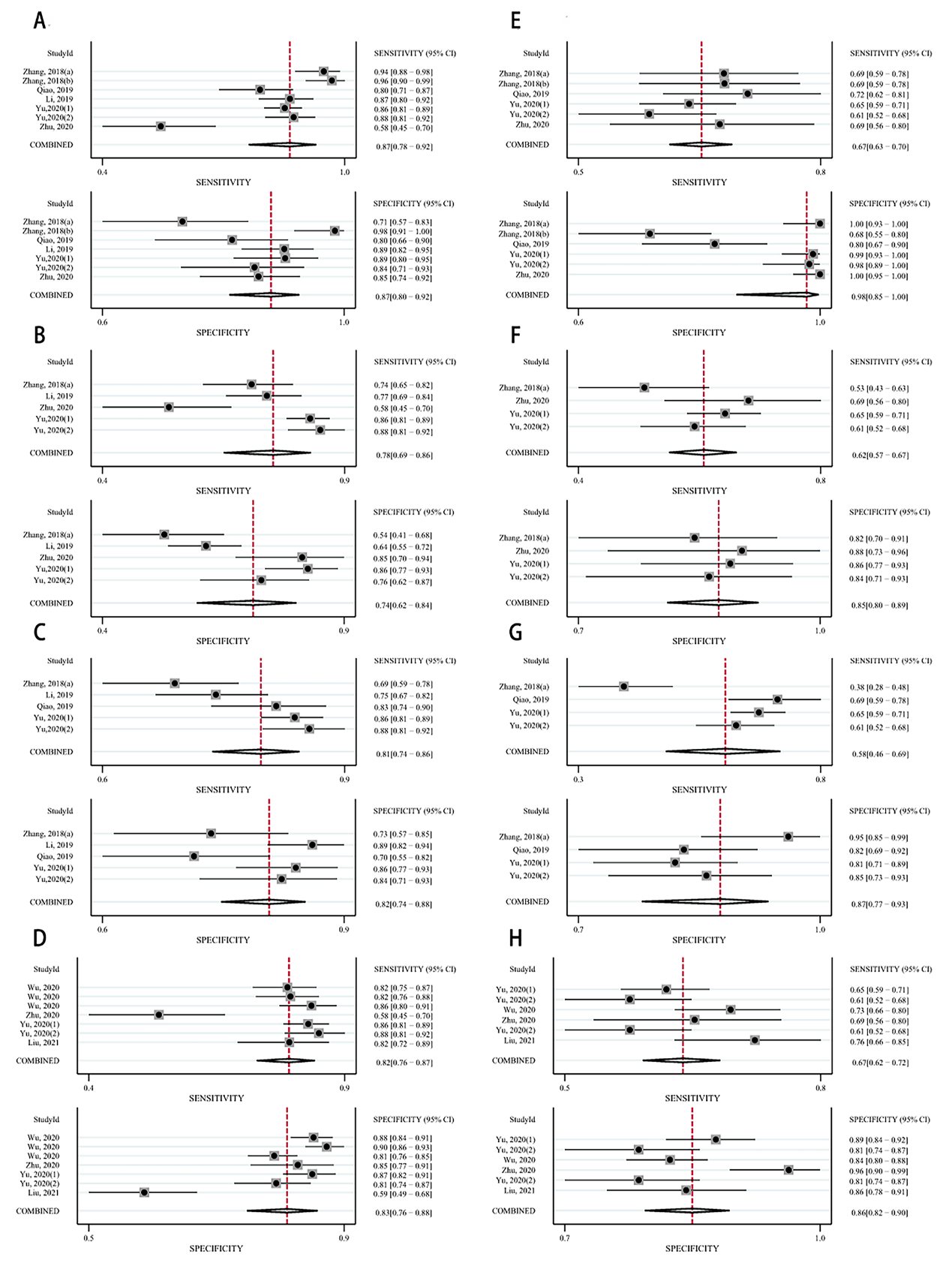
Supplementary Figure 2: Subgroup analysis of circRNAs and AFP.** Forests for (A) circRNAs in HCC patients vs Healthy controls; (B) circRNAs in HCC vs cirrhosis patients; (C) circRNAs in HCC vs Hepatitis patients; (D) circRNAs in HCC vs non-HCC patients; (E) AFP in HCC patients vs Healthy controls; (F) AFP in HCC vs cirrhosis patients; (G) AFP in HCC vs Hepatitis patients; (H) AFP in HCC vs non-HCC patients
