# Supplementary material for: Diagnostic Accuracy of Serum/Plasma Circular RNAs and the Combination of Circular RNAs and α-Fetoprotein for Detecting Hepatocellular Carcinoma: A Meta-Analysis
Source: Front Genet. 2021 Sep 30;12:722208. doi: 10.3389/fgene.2021.722208 (PMC8514948; doi:10.3389/fgene.2021.722208)
Supplement: Supplementary file 1 [file Data_Sheet_1.zip › Sup Table 1.DOCX]

| **Supplementary Table 2. List of studies about AFP included in the meta-analysis** | | | | | | | | | | | | | |
| --- | --- | --- | --- | --- | --- | --- | --- | --- | --- | --- | --- | --- | --- |
| **Study** | **Year** | **biomarker** | **Detection method** | **Study type** | **Sample type** | **Control type** | **Cut-off value** | **TP** | **FP** | **FN** | **TN** | **Sen** | **Spe** |
| **HCC vs Healthy** | | | | | | | | | | | | | |
| Zhang et al.(a) | 2018 | AFP | N/A | Case control study | plasma | HCC vs Healthy | 20 ng/ml | 72 | 0 | 32 | 52 | 0.694 | 1.000 |
| Qiao et al. | 2019 | AFP | N/A | Case control study | plasma | HCC vs Healthy | 400 ng/ml | 72 | 10 | 28 | 41 | 0.720 | 0.810 |
| Yu et al. | 2020 | AFP | N/A | Retrospective | plasma | HCC vs Healthy | 20 ng/ml | 189 | 1 | 101 | 75 | 0.652 | 0.987 |
|  |  | AFP | N/A | Retrospective | plasma | HCC vs Healthy | 20 ng/ml | 92 | 1 | 60 | 49 | 0.605 | 0.980 |
| Zhang et al.(b) | 2018 | AFP | ELISA | Case control study | serum | HCC vs Healthy | 23.5 ng/ml | 70 | 19 | 31 | 41 | 0.693 | 0.683 |
| Zhu et al. | 2020 | AFP | N/A | Retrospective | serum | HCC vs Healthy | 20 ng/ml | 44 | 0 | 20 | 72 | 0.688 | 1.000 |
| **HCC vs cirrhosis** | | | | | | | | | | | | | |
| Zhang et al.(a) | 2018 | AFP | N/A | Case control study | plasma | HCC vs cirrhosis | 20 ng/ml | 55 | 10 | 49 | 47 | 0.529 | 0.827 |
| Yu et al. | 2020 | AFP | N/A | Retrospective | plasma | HCC vs cirrhosis | 20 ng/ml | 189 | 11 | 101 | 69 | 0.652 | 0.863 |
|  |  | AFP | N/A | Retrospective | plasma | HCC vs cirrhosis | 20 ng/ml | 44 | 5 | 20 | 35 | 0.685 | 0.875 |
| Zhu et al. | 2020 | AFP | N/A | Retrospective | serum | HCC vs cirrhosis | 20 ng/ml | 92 | 8 | 60 | 42 | 0.605 | 0.840 |
| **HCC vs Hepatitis** | | | | | | | | | | | | | |
| Zhang et al.(a) | 2018 | AFP | N/A | Case control study | plasma | HCC vs HB | 20 ng/ml | 39 | 2 | 65 | 42 | 0.375 | 0.955 |
| Qiao et al. | 2019 | AFP | N/A | Case control study | plasma | HCC vs HB | 400 ng/ml | 69 | 9 | 31 | 42 | 0.690 | 0.830 |
| Yu et al. | 2020 | AFP | N/A | Retrospective | plasma | HCC vs HB | 20 ng/ml | 189 | 15 | 101 | 65 | 0.652 | 0.813 |
|  |  | AFP | N/A | Retrospective | plasma | HCC vs HB | 20 ng/ml | 92 | 8 | 60 | 46 | 0.605 | 0.852 |
| **HCC vs non-HCC** | | | | | | | | | | | | | |
| Liu et al. | 2021 | AFP | chemiluminescence immunoassay | Case control study | plasma | HCC vs nonHCC | 200 ng/ml | 68 | 17 | 21 | 102 | 0.764 | 0.857 |
| Yu et al. | 2020 | AFP | N/A | Retrospective | plasma | HCC vs nonHCC | 20 ng/ml | 189 | 27 | 101 | 209 | 0.652 | 0.886 |
|  |  | AFP | N/A | Retrospective | plasma | HCC vs nonHCC | 20 ng/ml | 92 | 29 | 60 | 125 | 0.605 | 0.812 |
| Zhu et al. | 2020 | AFP | N/A | Retrospective | serum | HCC vs nonHCC | 20 ng/ml | 44 | 5 | 20 | 107 | 0.688 | 0.955 |
| Wu et al. | 2020 | AFP | N/A | Retrospective | plasma | HCC vs nonHCC | N/A | 132 | 57 | 48 | 303 | 0.734 | 0.843 |
