# Supplementary material for: Diagnostic Accuracy of Serum/Plasma Circular RNAs and the Combination of Circular RNAs and α-Fetoprotein for Detecting Hepatocellular Carcinoma: A Meta-Analysis
Source: Front Genet. 2021 Sep 30;12:722208. doi: 10.3389/fgene.2021.722208 (PMC8514948; doi:10.3389/fgene.2021.722208)
Supplement: Supplementary file 1 [file Data_Sheet_1.zip › Sup Table 2.DOCX]

| **Supplementary Table 1. List of studies about circRNA included in the meta-analysis**  **Study Year biomarker Detection method Study type Sample type Control type Cut-off value TP FP FN TN Sen Spe**  **HCC vs Healthy**  **Zhang et al.(a) 2018 hsa_circ_0001445 qPCR Case control study plasma HCC vs Healthy N/A 98 15 6 37 0.942 0.712**  **Qiao et al. 2019 hsa_circ_0003998_ qPCR Case control study plasma HCC vs Healthy N/A 80 10 20 40 0.800 0.800**  **Yu et al. 2020 Circpanel qPCR Retrospective plasma HCC vs Healthy 0.5 248 8 42 68 0.855 0.895**  **Circpanel qPCR Retrospective plasma HCC vs Healthy 0.5 133 8 19 42 0.875 0.840**  **Li et al. 2019 circ SMARCA5 qPCR Case control study plasma HCC vs Healthy 15.28×10-2 117 11 18 92 0.867 0.893**  **Zhang et al.(b) 2018 circ_104075 qPCR Case control study serum HCC vs Healthy 1.66 97 1 4 59 0.960 0.983**  **Zhu et al. 2020 has_circ_0027089 qPCR Retrospective plasma HCC vs Healthy 0.011 37 11 27 61 0.578 0.847**  **HCC vs cirrhosis**  **Zhang et al.(a) 2018 hsa_circ_0001445 qPCR Case control study plasma HCC vs cirrhosis N/A 77 26 27 31 0.740 0.544**  **Yu et al. 2020 Circpanel qPCR Retrospective plasma HCC vs cirrhosis 0.5 248 11 42 69 0.855 0.863**  **Circpanel qPCR Retrospective plasma HCC vs cirrhosis 0.5 133 8 19 42 0.875 0.84**  **Li et al. 2019 circ SMARCA5 qPCR Case control study plasma HCC vs cirrhosis 10.34×10-2 104 52 31 91 0.770 0.636**  **Zhu et al. 2020 has_circ_0027089 qPCR Retrospective plasma HCC vs cirrhosis 0.011 37 6 27 34 0.578 0.850**  **HCC vs Hepatitis**  **Zhang et al.(a) 2018 hsa_circ_0001445 qPCR Case control study plasma HCC vs HB N/A 72 12 32 32 0.692 0.727**  **Qiao et al. 2019 hsa_circ_0003998_ qPCR Case control study plasma HCC vs HB N/A 83 15 17 35 0.830 0.700**  **Yu et al. 2020 Circpanel qPCR Retrospective plasma HCC vs HB 0.5 248 11 42 69 0.855 0.863**  **Circpanel qPCR Retrospective plasma HCC vs HB 0.5 133 8 19 42 0.875 0.759**  **Li et al. 2019 circ SMARCA5 qPCR Case control study plasma HCC vs HB+HC 9.385×10-2 101 13 34 104 0.748 0.889**  **HCC vs non-HCC**  **Liu et al. 2021 hsa_circ_0005397 qPCR Case control study plasma HCC vs nonHCC 0.914 73 49 16 70 0.820 0.588**  **Yu et al. 2020 Circpanel qPCR Retrospective plasma HCC vs nonHCC 0.5 248 11 42 69 0.855 0.863**  **Circpanel qPCR Retrospective plasma HCC vs nonHCC 0.5 133 29 19 125 0.875 0.812**  **Wu et al. 2020 circ_0009582 qPCR Retrospective plasma HCC vs nonHCC N/A 121 60 59 300 0.673 0.833**  **2020 circ_0037120 qPCR Retrospective plasma HCC vs nonHCC N/A 155 69 25 291 0.861 0.807**  **2020 circ_0140117 qPCR Retrospective plasma HCC vs nonHCC N/A 148 37 32 323 0.822 0.897**  **2020 Combination of circs qPCR Retrospective plasma HCC vs nonHCC N/A 147 45 33 315 0.817 0.875**  **Zhu et al. 2020 has_circ_0027089 qPCR Retrospective plasma HCC vs nonHCC 0.011 37 17 27 95 0.578 0.848. List of studies about circRNA included in the meta-analysis** | | | | | | | | | | | | | |
| --- | --- | --- | --- | --- | --- | --- | --- | --- | --- | --- | --- | --- | --- |
| **Study** | **Year** | **biomarker** | **Detection method** | **Study type** | **Sample type** | **Control type** | **Cut-off value** | **TP** | **FP** | **FN** | **TN** | **Sen** | **Spe** |
| **HCC vs Healthy** | | | | | | | | | | | | | |
| Zhang et al.(a) | 2018 | hsa_circ_0001445 | qPCR | Case control study | plasma | HCC vs Healthy | N/A | 98 | 15 | 6 | 37 | 0.942 | 0.712 |
| Qiao et al. | 2019 | hsa_circ_0003998_ | qPCR | Case control study | plasma | HCC vs Healthy | N/A | 80 | 10 | 20 | 40 | 0.800 | 0.800 |
| Yu et al. | 2020 | Circpanel | qPCR | Retrospective | plasma | HCC vs Healthy | 0.5 | 248 | 8 | 42 | 68 | 0.855 | 0.895 |
|  |  | Circpanel | qPCR | Retrospective | plasma | HCC vs Healthy | 0.5 | 133 | 8 | 19 | 42 | 0.875 | 0.840 |
| Li et al. | 2019 | circ SMARCA5 | qPCR | Case control study | plasma | HCC vs Healthy | 15.28×10^-2^ | 117 | 11 | 18 | 92 | 0.867 | 0.893 |
| Zhang et al.(b) | 2018 | circ_104075 | qPCR | Case control study | serum | HCC vs Healthy | 1.66 | 97 | 1 | 4 | 59 | 0.960 | 0.983 |
| Zhu et al. | 2020 | has_circ_0027089 | qPCR | Retrospective | plasma | HCC vs Healthy | 0.011 | 37 | 11 | 27 | 61 | 0.578 | 0.847 |
| **HCC vs cirrhosis** | | | | | | | | | | | | | |
| Zhang et al.(a) | 2018 | hsa_circ_0001445 | qPCR | Case control study | plasma | HCC vs cirrhosis | N/A | 77 | 26 | 27 | 31 | 0.740 | 0.544 |
| Yu et al. | 2020 | Circpanel | qPCR | Retrospective | plasma | HCC vs cirrhosis | 0.5 | 248 | 11 | 42 | 69 | 0.855 | 0.863 |
|  |  | Circpanel | qPCR | Retrospective | plasma | HCC vs cirrhosis | 0.5 | 133 | 8 | 19 | 42 | 0.875 | 0.84 |
| Li et al. | 2019 | circ SMARCA5 | qPCR | Case control study | plasma | HCC vs cirrhosis | 10.34×10^-2^ | 104 | 52 | 31 | 91 | 0.770 | 0.636 |
| Zhu et al. | 2020 | has_circ_0027089 | qPCR | Retrospective | plasma | HCC vs cirrhosis | 0.011 | 37 | 6 | 27 | 34 | 0.578 | 0.850 |
| **HCC vs Hepatitis** | | | | | | | | | | | | | |
| Zhang et al.(a) | 2018 | hsa_circ_0001445 | qPCR | Case control study | plasma | HCC vs HB | N/A | 72 | 12 | 32 | 32 | 0.692 | 0.727 |
| Qiao et al. | 2019 | hsa_circ_0003998_ | qPCR | Case control study | plasma | HCC vs HB | N/A | 83 | 15 | 17 | 35 | 0.830 | 0.700 |
| Yu et al. | 2020 | Circpanel | qPCR | Retrospective | plasma | HCC vs HB | 0.5 | 248 | 11 | 42 | 69 | 0.855 | 0.863 |
|  |  | Circpanel | qPCR | Retrospective | plasma | HCC vs HB | 0.5 | 133 | 8 | 19 | 42 | 0.875 | 0.759 |
| Li et al. | 2019 | circ SMARCA5 | qPCR | Case control study | plasma | HCC vs HB+HC | 9.385×10^-2^ | 101 | 13 | 34 | 104 | 0.748 | 0.889 |
| **HCC vs non-HCC** | | | | | | | | | | | | | |
| Liu et al. | 2021 | hsa_circ_0005397 | qPCR | Case control study | plasma | HCC vs nonHCC | 0.914 | 73 | 49 | 16 | 70 | 0.820 | 0.588 |
| Yu et al. | 2020 | Circpanel | qPCR | Retrospective | plasma | HCC vs nonHCC | 0.5 | 248 | 11 | 42 | 69 | 0.855 | 0.863 |
|  |  | Circpanel | qPCR | Retrospective | plasma | HCC vs nonHCC | 0.5 | 133 | 29 | 19 | 125 | 0.875 | 0.812 |
| Wu et al. | 2020 | circ_0009582 | qPCR | Retrospective | plasma | HCC vs nonHCC | N/A | 121 | 60 | 59 | 300 | 0.673 | 0.833 |
|  | 2020 | circ_0037120 | qPCR | Retrospective | plasma | HCC vs nonHCC | N/A | 155 | 69 | 25 | 291 | 0.861 | 0.807 |
|  | 2020 | circ_0140117 | qPCR | Retrospective | plasma | HCC vs nonHCC | N/A | 148 | 37 | 32 | 323 | 0.822 | 0.897 |
|  | 2020 | Combination of circs | qPCR | Retrospective | plasma | HCC vs nonHCC | N/A | 147 | 45 | 33 | 315 | 0.817 | 0.875 |
| Zhu et al. | 2020 | has_circ_0027089 | qPCR | Retrospective | plasma | HCC vs nonHCC | 0.011 | 37 | 17 | 27 | 95 | 0.578 | 0.848 |
