# Supplementary material for: Diagnostic Accuracy of Serum/Plasma Circular RNAs and the Combination of Circular RNAs and α-Fetoprotein for Detecting Hepatocellular Carcinoma: A Meta-Analysis
Source: Front Genet. 2021 Sep 30;12:722208. doi: 10.3389/fgene.2021.722208 (PMC8514948; doi:10.3389/fgene.2021.722208)
Supplement: Supplementary file 1 [file Data_Sheet_1.zip › Sup Table 3.DOCX]

| **Supplementary Table 3. List of studies about combination of AFP and circRNA included in the meta-analysis** | | | | | | | | | | | |
| --- | --- | --- | --- | --- | --- | --- | --- | --- | --- | --- | --- |
| **Study** | **Year** | **biomarker** | **Study type** | **Sample type** | **Control type** | **TP** | **FP** | **FN** | **TN** | **Sen** | **Spe** |
| **HCC vs Healthy** | | | | | | | | | | | |
| Zhang et al.(a) | 2018 | Combination | Case control study | plasma | HCC vs Healthy | 91 | 3 | 13 | 49 | 0.875 | 0.942 |
| Qiao et al. | 2019 | Combination | Case control study | plasma | HCC vs Healthy | 88 | 4 | 12 | 46 | 0.880 | 0.920 |
| Yu et al. | 2020 | Combination | Retrospective | plasma | HCC vs Healthy | 266 | 8 | 24 | 68 | 0.917 | 0.895 |
|  |  | Combination | Retrospective | plasma | HCC vs Healthy | 141 | 8 | 11 | 42 | 0.928 | 0.840 |
| Li et al. | 2019 | Combination | Case control study | plasma | HCC vs Healthy | 135 | 0 | 0 | 103 | 1.000 | 1.000 |
| Zhang et al.(b) | 2018 | Combination | Case control study | serum | HCC vs Healthy | 51 | 10 | 13 | 62 | 0.797 | 0.861 |
| Zhu et al. | 2020 | Combination | Retrospective | serum | HCC vs Healthy | 141 | 8 | 11 | 42 | 0.928 | 0.840 |
| **HCC vs cirrhosis** | | | | | | | | | | | |
| Zhang et al.(a) | 2018 | Combination | Case control study | plasma | HCC vs cirrhosis | 65 | 12 | 39 | 45 | 0.625 | 0.788 |
| Yu et al. | 2020 | Combination | Retrospective | plasma | HCC vs cirrhosis | 266 | 14 | 24 | 66 | 0.917 | 0.825 |
|  |  | Combination | Retrospective | plasma | HCC vs cirrhosis | 141 | 9 | 11 | 41 | 0.928 | 0.820 |
| Li et al. | 2019 | Combination | Case control study | plasma | HCC vs cirrhosis | 97 | 24 | 38 | 119 | 0.719 | 0.832 |
| Zhu et al. | 2020 | Combination | Retrospective | serum | HCC vs cirrhosis | 51 | 10 | 13 | 30 | 0.797 | 0.750 |
| **HCC vs Hepatitis** | | | | | | | | | | | |
| Zhang et al.(a) | 2018 | Combination | Case control study | plasma | HCC vs HB | 84 | 8 | 20 | 36 | 0.808 | 0.818 |
| Qiao et al. | 2019 | Combination | Case control study | plasma | HCC vs HB | 73 | 11 | 27 | 39 | 0.730 | 0.780 |
| Yu et al. | 2020 | Combination | Retrospective | plasma | HCC vs HB | 266 | 18 | 24 | 62 | 0.917 | 0.775 |
|  |  | Combination | Retrospective | plasma | HCC vs HB | 141 | 14 | 11 | 40 | 0.928 | 0.741 |
| Li et al. | 2019 | Combination | Case control study | plasma | HCC vs HB+HC | 105 | 12 | 30 | 105 | 0.778 | 0.897 |
| **HCC vs nonHCC** | | | | | | | | | | | |
| Liu et al. | 2021 | Combination | Case control study | plasma | HCC vs nonHCC | 80 | 51 | 9 | 68 | 0.899 | 0.571 |
| Yu et al. | 2020 | Combination | Retrospective | plasma | HCC vs nonHCC | 266 | 40 | 24 | 196 | 0.917 | 0.831 |
|  |  | Combination | Retrospective | plasma | HCC vs nonHCC | 141 | 31 | 11 | 123 | 0.928 | 0.799 |
| Zhu et al. | 2020 | Combination | Retrospective | serum | HCC vs nonHCC | 51 | 20 | 13 | 92 | 0.797 | 0.821 |
| Wu et al. | 2020 | Combination | Retrospective | plasma | HCC vs nonHCC | 168 | 4 | 12 | 356 | 0.935 | 0.988 |
